# A multi-sensor dataset with annotated activities of daily living recorded in a residential setting

**DOI:** 10.1038/s41597-023-02017-1

**Published:** 2023-03-23

**Authors:** Emma L. Tonkin, Michael Holmes, Hao Song, Niall Twomey, Tom Diethe, Meelis Kull, Miquel Perello Nieto, Massimo Camplani, Sion Hannuna, Xenofon Fafoutis, Ni Zhu, Przemysław R. Woznowski, Gregory J. L. Tourte, Raúl Santos-Rodríguez, Peter A. Flach, Ian Craddock

**Affiliations:** 1 University of Bristol, Bristol, UK; 2 Amazon, Bellevue, USA; 3 University of Tartu, Tartu, Estonia; 4 Technical University of Denmark, Lyngby, Denmark; 5 China Mobile International, Beijing, China; 6 BJSS, Manchester, UK

**Keywords:** Quality of life, Electrical and electronic engineering, Scientific data

## Abstract

SPHERE is a large multidisciplinary project to research and develop a sensor network to facilitate home healthcare by activity monitoring, specifically towards activities of daily living. It aims to use the latest technologies in low powered sensors, internet of things, machine learning and automated decision making to provide benefits to patients and clinicians. This dataset comprises data collected from a SPHERE sensor network deployment during a set of experiments conducted in the ‘SPHERE House’ in Bristol, UK, during 2016, including video tracking, accelerometer and environmental sensor data obtained by volunteers undertaking both scripted and non-scripted activities of daily living in a domestic residence. Trained annotators provided ground-truth labels annotating posture, ambulation, activity and location. This dataset is a valuable resource both within and outside the machine learning community, particularly in developing and evaluating algorithms for identifying activities of daily living from multi-modal sensor data in real-world environments. A subset of this dataset was released as a machine learning competition in association with the European Conference on Machine Learning (ECML-PKDD 2016).

## Background & summary

Obesity, depression, stroke, falls, cardiovascular and musculoskeletal diseases are some of the most significant health issues and fastest-rising categories of healthcare costs. The financial expenditure associated with these conditions is widely regarded as ineffective and unsustainable. The impact on quality of life is also felt by millions of people around the world each day.

Smart technologies can unobtrusively quantify activities of daily living, and these can provide long-term behavioural patterns that are objective, insightful measures for clinical professionals and caregivers. The EPSRC-funded Sensor Platform for HEalthcare in Residential Environment (SPHERE) Interdisciplinary Research Collaboration (IRC)^1–3^ project has designed a multi-modal sensor system driven by data analytics requirements. The system is currently under test in over 50 homes across Bristol (UK). The data sets collected will be made available to researchers in a variety of communities. This paper describes a particular dataset focusing on the task of activity recognition using machine learning approaches.

To collect this dataset, the overall sensing system includes the following three sensing modalities:

- wrist-worn accelerometer;
- RGB-D cameras (video with depth information); and
- passive environmental sensors.

With these sensors, the system is capable of capturing information related to most indoor daily living activities. It is then possible to learn patterns of behaviour and track the deterioration/recovery of persons that suffer or recover from various medical conditions. To achieve this design, sensor data in this dataset is also accompanied by annotations of domestic human behaviour to facilitate classification of:

- activities of daily living (tasks such as meal preparation, watching television);
- posture/ambulation (e.g., walking, sitting, transitioning); and
- room-level indoor location.

On the machine learning side, these data have value in training, optimising and evaluating activity recognition and localisation classification algorithms, as well as generic time series models. The data can also be used as a source dataset for exploring sensor fusion methods and multi-modal learning approaches. For example, these data have been used in *The SPHERE Challenge*^4^ competition by students and researchers to model and classify activities of daily living, and the wining approach is documented by Liu *et al*.^5^. In a related paper^6^, the authors propose an unsupervised approach to learn the sensor layout for a multi-modal sensor network within a residential environment. The overall ML system construction and applications are discussed for the SPHERE project^7^. On the healthcare side, these data support further development of methods for Smart Home and e-health applications. Recent research shows that the classification of natural human movement can indicate the recovery level from hip and knee replacement surgery^8^.

The remainder of this paper is structured as follows: Methods describes the methods used to collect these data. Data Records. provides detail on the data records, structures and format, Technical Validation describes the method and results of technical validation and finally, Usage Notes highlights notes for use of these data. Code availability includes details on code availability.

## Methods

The following section describes recruitment, data collection, data extraction and processing for this dataset, as well as the validation processes undergone for label annotations.

### Recruitment

Ten healthy volunteer participants (two female, eight male) were recruited from the student and faculty community at the University of Bristol to participate in a data collection activity. Eight participants were in the 18–29 category, two in 30–39. Participants filled out a health questionnaire prior to participation, and no health issues were recorded.

**Fig. 1:**
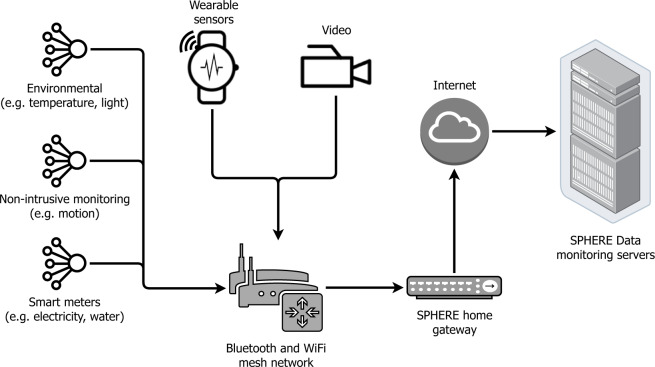
The SPHERE sensor network. Note that the environmental and smart meter data are excluded from this dataset, as they are not relevant to the scripted tasks.

**Fig. 2:**
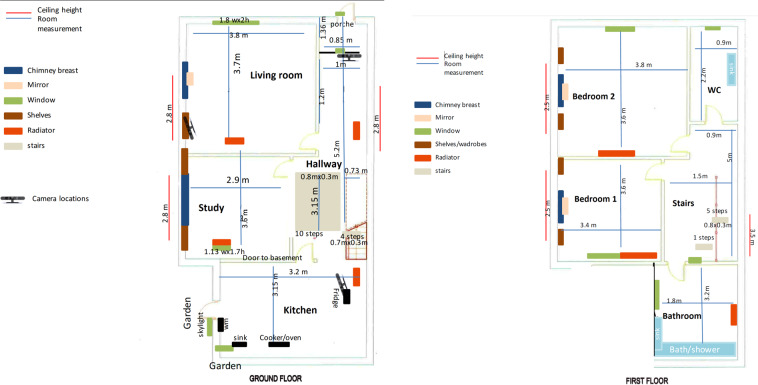
The floorplan of the SPHERE smart home. (**Left**) Ground floor. (**Right**) First floor.

The data was collected in the SPHERE house (Fig. 2), a test-bed property in Bristol, UK, which has been fitted with the SPHERE sensor network (Fig. 1). Ethical approval was secured from the University of Bristol’s ethics committee to conduct data collection, and informed consent was obtained from all volunteers. Volunteers were not compensated for participation.

### Data collection

Data were collected within the SPHERE House. All recording sessions occurred in timeslots between 10 am and 4 pm over ten working days in the late summer (Aug–Oct). Overall length of session ranged from 23 min 57 s to 36 min 46 s, mean 00:29:20, stdev 00:02:59. Time of day of data collection has been excluded from the dataset as a participant privacy measure. Figure 2 show the floor plan of the ground and first floors of the smart environment respectively. The SPHERE House is designed to be a unique environment with controllable experimental conditions. Valuable smart-home data can be collected on multiple participants without the prohibitive time and cost burdens associated with installation and removal of a large number of sensors in multiple residential locations.

Figure 1 shows the SPHERE sensor network with constellations of sensors sending measurements back to the Home Gateway. The SPHERE sensor network incorporates multiple modalities of data: environmental, video and wearable-sensor streams. Data from sensors were streamed via MQTT to a Mongo database on the Home Gateway. All sensors are synchronised with network time protocol (NTP). Following experimentation, data from the home gateway database was exported for annotation and analysis.

As introduced above, the dataset captures scripted activities performed by multiple participants – one participant at a time – within the SPHERE house. The experimental script for activities can be found at https://raw.githubusercontent.com/IRC-SPHERE/sphere-challenge/master/documents/data_collection_script.pdf. The script includes behaviours and activities such as room and floor transitions, posture and ambulation changes, interaction with domestic appliances and simulated activities of daily living. Each participant undertook activities individually with the supervision of a SPHERE project researcher. While the order of activities was enforced, the pace is not scheduled and could be different for every participant.

### Data extraction and processing

The following subsections describe the sensing modalities that are found in the smart home and explain the overall data extraction and processing steps.

#### Accelerometers

Participants wore the SPHERE wearable on their dominant wrist (the device used was the first generation SPW-1, as described by Fafoutis *et al*.^9^), attached using a strap. The SPHERE wearable is an acceleration-based activity sensor. The device is equipped with two ADXL362 accelerometers^10^ and wirelessly transmits data using the Bluetooth Low Energy (BLE) standard to several access points (receivers) positioned within the house^11^. The outputs of these sensors are a continuous numerical stream of the accelerometer readings (units of *g*, i.e., approximately 9.81 m s^−2^). Accompanying the accelerometer readings are the received signal strength indications (RSSI) that were recorded by each access point (in units of dBm), and these data will be informative for indoor localisation. The accelerometers record data at 20 Hz (12-bit resolution), and the accelerometer ranges are between ±8 *g*. RSSI values are also recorded at 20 Hz, and values are no lower than −110 dBm.

For privacy concerns, all the residential environments are assumed to be hard-to-access, hence should require minimum to no maintenance for long-term operations. Therefore, the system is optimised for low energy consumption. To that end, the communication between the wearable sensor and the smart house is performed via undirected connectionless BLE advertisements. Although data reliability can be addressed at the receiver^11^, this communication approach does not provide delivery guarantees and, thus, there may be missing packets from the data. Recent accelerometer work done by SPHERE researchers on activity recognition with accelerometers includes^12,13^. Data from the SPHERE wearable have also been used for the validation of a privacy-preserving algorithm for wearable embedded systems^14^.

#### RGB-D cameras

Video recordings were taken using ASUS Xtion PRO RGB-Depth (RGB-D) cameras. Automatic detection of humans was performed using the OpenNI library, and false-positive detections were manually removed by visual inspection. Three RGB-D cameras are installed in the SPHERE house, and these are located in the living room, hallway, and the kitchen. No cameras are located elsewhere in the house due to privacy considerations.

In order to preserve the anonymity of the participants, the raw video data are not stored. Instead, several data points are stored describing the detected individual: the coordinates of the 2D bounding box enclosing them, the 2D centre of mass, the 3D bounding box enclosing the individual and the 3D centre of mass are collected into the dataset. These are generated using OpenNI and OpenCV on the basis of RGB-D data. The limitations of this data include occlusion (e.g., if a participant is partially occluded by a wall or other object, the bounding box will be truncated) and a risk of false-negative or false-positive detection. Imperfect detection of object boundaries is also a possibility. Occlusion was minimised by the experimental script and by a careful choice of recording angles. Data was screened for missing modalities or false positives.

The units of 2D coordinates are in pixels (i.e., number of pixels down and right from the upper left-hand corner) from an image of size 640 × 480 pixels. The coordinate system of the 3D data is axis-aligned with the 2D bounding box, with an extra dimension that projects from the central position of the video frames. The first two dimensions specify the vertical and horizontal displacement of a point from the central vector (in millimetres), and the final dimension specifies the projection of the object along the central vector (again, in millimetres).

RGB-D tracking data is very valuable in indoor environments as it can facilitate improvement in accuracy for fundamental computer vision tasks such as tracking^15^ while also enabling specific analysis at higher levels. RGB-D data collected in the SPHERE house has been used for example for specific action recognition^16^ and action quality estimation^17^.

#### Environmental sensors

The environmental sensing nodes are built on development platforms (Libelium, with CE marking), powered by batteries or/and 5 V DC converted from mains. Passive Infra-Red (PIR) sensors are employed to detect presence in the data. Values of 1 indicate that motion was detected, whereas values of 0 mean that no motion was detected. Several methods that deal with environmental sensor data are detailed in the following^6,18^.

### Annotation

The annotation processes use a simplified taxonomy based on the SPHERE annotation ontology^19^. This ontology was developed with reference to various existing sources, notably the taxonomy published by BoxLab. A team of 12 annotators was recruited and trained to annotate the set of locations and activities (Table 1). Every data sequence was annotated by either 2 or 3 annotators to avoid bias in the ground truth. Four sessions were annotated by three annotators. As the pairwise Cohen’s kappa calculated remained reasonably stable between annotators in most cases, the decision was taken to reduce the number of annotators to two for the remaining sessions (see Table 3). To support the annotation process, a head-mounted camera (Panasonic HX-A500E-K 4 K Wearable Action Camera Camcorder) recorded 4 K video at 25 FPS to an SD-card. This data is only used to facilitate the annotation process and is removed after the annotation and therefore is not shared in this dataset. Synchronisation between the NTP clock and the head-mounted camera was achieved by focusing the camera on an NTP-synchronised digital clock at the beginning and end of the recording sequences. An annotation tool called ELAN^20^ was used for annotation. ELAN is a tool for the creation of complex annotations on video and audio resources, developed by the Max Planck Institute for Psycholinguistics in Nijmegen, The Netherlands.

**Table 1:** Annotation labels for locations, activities, postures and transitions.

Type	Labels
Locations	bath, bed1, bed2, hall, kitchen, living, stairs, study, toilet
Ambulation	a_ascend, a_descend, a_jump, a_loadwalk, a_walk
Posture	p_bent, p_kneel, p_lie, p_sit, p_squat, p_stand
Transition	t_bend, t_kneel_stand, t_lie_sit, t_sit_lie, t_sit_stand, t_stand_kneel, t_stand_sit, t_straighten, t_turn

**Fig. 3:**
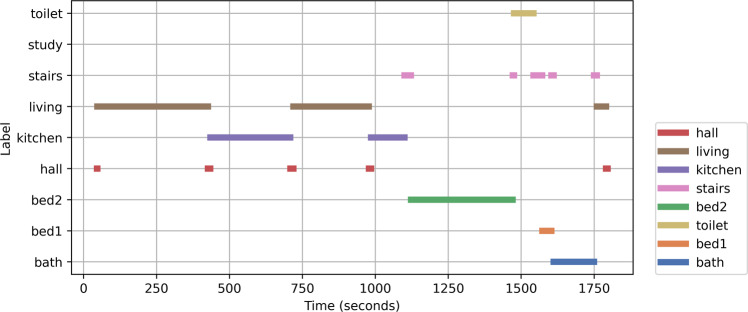
An example of annotated locations for one participant, with time shown in seconds.

Annotators labelled the data set to indicate the location of the participant, using the labels shown in Table 1 (note that only one bedroom is in use for this experiment). Figure 3 shows location annotations for one participant.

In addition to location, ambulation (e.g., walking, climbing the stairs), posture (e.g., sitting, standing, lying), transitions between postures, activity (e.g., brushing teeth, preparing a toast with jam and a cup of tea) annotations involving actions using the participant’s hands were also recorded, in particular: using, holding, grabbing and releasing an object for both left and right hand. Alongside scripted activities, participants were also asked to complete a brief set of jumps to ensure synchronisation of wearable data. The annotations are defined in Table 2.

**Table 2:** Ontology definitions and detailed description of the activity labels found in the dataset.

Label	Ontology Definition	Description
***Ambulation***		
a_ascend	ascending stairs	Ascending stairs
a_descend	descending stairs	Descending stairs
a_jump	jumping	A standing vertical jump
a_walk	walking	Walking without objects in one’s hands
a_loadwalk	Walking with load	walking whilst holding objects (e.g. a bowl).
***Posture***		
p_bent	bend over	Static stand with the torso bent forward at any angle.
p_kneel	kneeling	Crouching with knees bent, resting on the knees.
p_lie	lying	Lying down in any position (e.g. supine, prone)
p_sit	sitting on chair/bed/sofa	Any seated posture other than those defined elsewhere.
p_squat	squating	Crouching with knees bent and balancing on the feet
p_stand	standing	Any standing posture.
***Transition***		
t_bend	bending	Bending the torso forward, defined to include partial rotation.
t_straighten	straightening	Straightening the torso.
t_turn	turning/pivoting	A turn or pivot of the torso, defined to include partial rotation.
t_stand_kneel	stand to kneel	Transition from a standing position to a kneeling position.
t_kneel_stand	kneel to stand	Transition from a kneeling position to a standing position.
t_lie_sit	lying to sit	Transition from lying down (typically in a supine position) to a standing position
t_sit_lie	sit to lying	Transition from a seated position to lying down.
t_sit_stand	sit to stand	Transition from a seated position to a standing position.
t_stand_sit	stand to sit	Transition from a standing position to a seated position.

**Fig. 4:**
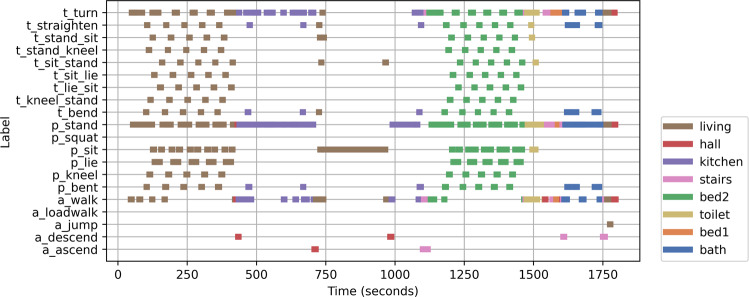
An example of annotated activities in locations for one participant, with time shown in seconds.

Posture transitions were performed in groups of 5, e.g., stand-sit-lay-sit-stand performed by a participant 5 times in a row. Table 1 shows a list of all activity annotation labels. Figure 4 shows annotated activities in locations for one participant.

The annotation process resulted in millisecond-accuracy labels that are described by two timestamps: one for the start time and one for the end time. For a typical supervised learning setting, these labels are transformed into probabilistic labels for each one-second time window. For any time window that contains only one label, we assign a probability vector with one on the corresponding label and zero elsewhere.

In contrast, if a time window contains more than one type of label, we calculate the time length of each label, from which we obtain the relative proportion of each label, and assign it as the resulted probability vector. While such an approach can naturally deal with multiple labels within a given time window, it can be further used to solve disagreements among multiple annotators. We can use the time length annotated by each annotator to calculate the final proportion and probability vector. Figure 4 shows the annotated activities in locations for one participant.

Interrater reliability was calculated using Cohen’s kappa^21^, using the implementation provided in the scikit-learn package. Table 3 shows per-annotation, per-annotator-pair scores calculated across all sessions in which two or more annotation sets were available. Across all annotations, the majority of pairs achieved moderate agreement as defined by Cohen and McHugh^22^ (pairwise mean > 0.61). However, performance varied significantly on individual annotations. Some annotations were employed inconsistently, notably ‘walking with load (loadwalk)’, the posture ‘squatting’ and the location ‘toilet’, which are seldom used; this results in low agreement (loadwalk $\bar{\kappa } > 0.22$; squatting: $\bar{\kappa } > 0.47$; toilet: $\bar{\kappa } > 0.33$. There are also inconsistencies in the annotations for bedroom, where some differentiate between the different bedrooms, and other do not. For the purpose of the interrater table (Table 3), the different bedroom labels have been merged into a single ‘bedroom’ label. There is fair agreement on turns ($\bar{\kappa } > 0.39$) and moderate agreement on bending ($\bar{\kappa } > 0.54$), which may reflect the brief duration of these actions (average duration 1.09 s in both cases); difficulty in annotating turns may also result from the use of a head-mounted camera, and could potentially be reduced by supplementary information from additional, fixed cameras. All other annotations see moderate, substantial or in some cases almost perfect agreement.

**Table 3:** Interrater reliability: Pairwise Cohen’s Kappas calculated per annotation for all sessions. Over all annotations, the majority of pairs (11 out of 18) achieved moderate agreement.

Session	1			2			3	4			5	6	7	8	9			10	$\bar{k}$	Usage per session	*σ* (usage)	Average duration (s)	*σ* (s)
Annotator pairs	A-B	A-C	B-C	D-E	D-F	E-F	G-H	I-J	I-K	J-K	L-M	N-O	P-Q	R-S	T-U	T-V	U-V	X-Y					
Ambulation: descending stairs	0.80	0.78	0.93	0.76	0.71	0.77	0.85	0.69	0.78	0.57	0.95	0.63	0.87	0.81	0.73	0.87	0.85	0.94	**0.79**	7.50	3.22	3.35	1.22
Ambulation: ascending stairs	0.90	0.89	0.86	0.81	0.74	0.82	0.91	0.72	0.93	0.78	0.92	0.91	0.85	0.75	0.80	0.90	0.86	0.60	**0.81**	6.19	3.29	4.39	3.55
Ambulation: jumping	0.89	0.91	0.8	0.67	0.4	0.67	0.77	0.75	1.0	0.75	0.73	0.8	1.0	0.67	1.0	0.83	0.83	1.0	**0.80**	4.19	2.20	1.99	0.67
Ambulation: walking with load	1.0	0.0	0.0	0.0	1.0	0.0	1.0	0.47	0.0	0.0	0.0	0.0	0.0	0.0	0.0	0.0	0.56	0.0	**0.22**	5.44	5.70	3.58	2.80
Ambulation: walking	0.72	0.65	0.65	0.52	0.76	0.6	0.67	0.63	0.53	0.56	0.61	0.67	0.58	0.62	0.61	0.65	0.64	0.57	**0.62**	116.38	54.21	2.38	1.56
Posture: bent over	0.67	0.73	0.79	0.71	0.66	0.57	0.72	0.65	0.79	0.71	0.76	0.79	0.82	0.53	0.46	0.57	0.68	0.45	**0.67**	40.94	20.12	3.37	5.07
Posture: kneeling	0.73	0.43	0.44	0.53	0.65	0.66	0.83	0.34	0.52	0.46	0.72	0.89	0.88	0.23	0.66	0.73	0.79	0.82	**0.63**	15.00	9.01	3.36	0.60
Posture: lying	0.91	0.91	0.92	0.85	0.84	0.86	0.83	0.91	0.82	0.87	0.85	0.93	0.73	0.75	0.91	0.9	0.92	0.91	**0.87**	50.56	21.18	4.82	1.52
Posture:sitting on chair bed sofa	0.89	0.71	0.78	0.89	0.13	0.12	0.93	0.18	0.84	0.25	0.87	0.91	0.75	0.76	0.89	0.85	0.93	0.91	**0.7**	46.00	21.34	11.89	33.60
Posture: squatting	0.0	0.0	0.43	0.0	0.0	−0.01	1.0	0.0	1.0	0.0	1.0	1.0	1.0	0.0	1.0	1.0	1.0	0.0	**0.47**	2.81	3.21	3.83	0.85
Posture: standing	0.82	0.82	0.82	0.75	0.8	0.74	0.83	0.71	0.77	0.78	0.82	0.87	0.83	0.79	0.76	0.79	0.81	0.79	**0.79**	204.75	94.77	4.95	7.34
Transition: lying to sit	0.8	0.76	0.8	0.52	0.86	0.66	0.62	0.61	0.61	0.88	0.82	0.86	0.55	0.24	0.88	0.86	0.9	0.72	**0.72**	20.13	8.55	1.61	0.51
Transition: sit to lying	0.81	0.72	0.68	0.75	0.72	0.7	0.9	0.72	0.65	0.68	0.49	0.8	0.53	0.33	0.86	0.77	0.82	0.75	**0.7**	19.94	8.50	1.58	0.51
Transition: kneel to stand	0.76	0.36	0.35	0.51	0.62	0.66	0.68	0.51	0.45	0.33	0.71	0.93	0.74	0.15	0.72	0.68	0.78	0.8	**0.6**	15.00	9.18	1.40	0.30
Transition: Stand to kneel	0.72	0.27	0.22	0.46	0.54	0.58	0.9	0.35	0.6	0.26	0.73	0.75	0.8	0.0	0.51	0.43	0.87	0.68	**0.54**	15.56	8.78	1.68	0.46
Transition: sit to stand	0.68	0.74	0.68	0.37	0.51	0.47	0.69	0.64	0.47	0.6	0.83	0.66	0.74	0.66	0.68	0.6	0.71	0.51	**0.62**	25.31	11.76	1.29	0.46
Transition: stand to sit	0.73	0.55	0.55	0.57	0.55	0.45	0.64	0.59	0.59	0.71	0.68	0.79	0.77	0.64	0.66	0.63	0.7	0.66	**0.64**	25.38	11.63	1.52	0.65
Transition: bending	0.6	0.5	0.4	0.39	0.52	0.51	0.7	0.48	0.48	0.51	0.52	0.78	0.6	0.51	0.54	0.54	0.56	0.54	**0.54**	47.69	26.47	1.09	0.48
Transition: straightening	0.7	0.58	0.69	0.39	0.65	0.46	0.65	0.56	0.59	0.61	0.46	0.72	0.61	0.54	0.53	0.58	0.58	0.53	**0.58**	46.75	25.22	1.08	0.37
Transition: turning pivoting	0.55	0.56	0.52	0.36	0.3	0.27	0.42	0.25	0.31	0.28	0.4	0.55	0.45	0.41	0.4	0.38	0.18	0.41	**0.39**	313.63	163.27	1.19	0.60
Bathroom 0.8	1.0	0.81	0.78	1.0	0.78	0.77	0.75	0.75	0.99	0.8	1.0	0.81	0.82	0.99	0.82	0.83	0.8	0.85	**3.0**	3.00	1.37	101.28	54.86
Bedroom	1.0	1.0	1.0	0.99	0.99	1.0	1.0	1.0	1.0	1.0	1.0	1.0	0.86	1.0	0.99	1.0	1.0	1.0	**0.99**	3.63	1.71	166.02	149.78
Hallway	0.78	0.58	0.59	0.9	0.88	0.95	0.44	0.86	0.87	0.92	0.88	0.88	0.77	0.93	0.88	0.89	0.93	0.94	**0.83**	18.38	7.96	13.72	37.41
Kitchen	1.0	0.99	0.99	0.98	0.98	1.0	0.72	0.99	0.99	0.99	1.0	0.99	0.99	0.99	0.99	0.99	1.0	0.99	**0.98**	3.75	1.61	158.11	63.02
Living room	0.99	1.0	0.99	1.0	1.0	1.0	1.0	0.99	0.99	0.99	1.0	1.0	1.0	0.69	1.0	1.0	1.0	1.0	**0.98**	5.75	2.79	212.02	126.14
Toilet	0.0	0.0	1.0	0.0	0.98	0.0	0.0	0.0	0.0	1.0	0.0	0.96	1.0	0.0	1.0	0.0	0.0	0.0	**0.33**	0.81	0.75	53.3	0.0
**Pairwise mean**	**0.72**	**0.63**	**0.66**	**0.57**	**0.64**	**0.56**	**0.71**	**0.55**	**0.63**	**0.6**	**0.68**	**0.77**	**0.72**	**0.52**	**0.71**	**0.67**	**0.7**	**0.64**					

## Data Records

The SPHERE multi-sensor dataset is published as an openly available set of CSV files at the University of Bristol institutional data repository, with the DOI: 10.5523/bris.2h0wyctxrd69j2oqccsi45hy1p^23^. This dataset is available under the CC-BY licence. This section outlines the format of the data records available in this dataset.

### Directory structure

This dataset provides three views of the data: raw, sphere-challenge-2016 and sphere-challenge-complete-2022, each of which can be found under the data directory. The raw view consists of the full, complete and un-shuffled data (in interval and tabular form) and labels. sphere-challenge-2016 is a replication of the SPHERE Challenge that took part in ECML 2016^4^, but with more consistent cross-modality naming conventions and activity/location labels of the test set. Train and test data are created in such a way that every user from the test set can be found in the train set. The data for this view was shuffled and randomly sliced in order to force the participants of the challenge to learn activity and location recognition models rather than memorising the script. Finally, the sphere-challenge-complete-2022 has preserved the train and test splits from the SPHERE Challenge and the sequences are provided in un-sliced format. This means there are fewer sequences in this view, but each sequence is long (approximately 30 mins). The data provided in the sphere-challenge-2016 and sphere-challenge-complete-2022 views are provided in dense, CSV format (i.e., raw interval data are not provided), and the code that produces these views can be found in main.py.

Taking the sphere-challenge-2016 view, two sub-folders, for the training set and testing set respectively can be found. The training set contains 10 folders with long sequences of recorded data, with each sequence lasting around 20 to 30 minutes. The testing set contains 872 folders with short sequences of recorded data, and most sequences are about 30 seconds.

All recorded data are marked with unique codes (each recording will be referred to as a ‘sequence folder’). Timestamps are re-based to be relative to the start of the sequences, i.e., each sequence always starts from *t* = 0. However, individual CSV files begin with an offset that reflects the time between the timestamp at which the sequence began and the datetime at which the first subsequent data point was generated: for example, accelerometer data may begin some milliseconds following *t* = 0, while video data, which is event-driven, may begin long after *t* = 0 or not appear in a certain sample at all.

Each sequence folder contains the following files for sensor data:

- pir.csv and/or pir_raw.csv
- acceleration.csv
- rssi.csv
- rgbd_hall.csv
- rgbd_living.csv
- rgbd_kitchen.csv
- meta.json

Each folder furthermore contains a set of files for the annotations with the following name formats:

- activity.csv
- location.csv
- per_ann_activity_*.csv and/or activity_*_raw.csv
- per_ann_location_*.csv and/or location_*_raw.csv

Here * will be a positive integer indicating corresponding annotator. That is, having two files as per_ann_activity_1.csv and per_ann_activity_2.csv means there are two different annotators for the activity labels. The activity.csv and location.csv files contain the merged ground-truth from all annotators.

### Sensor data files

#### pir_raw.csv

This file contains the start time and duration for all PIR sensors in the smart environment. PIR sensors are located in the following nine rooms: *bath*, *bed1*, *bed2*, *hall*, *kitchen*, *living*, *stairs*, *study*, *toilet*. The columns of this CSV file are:

- **start**: the start time of the PIR sensor (relative to the start of the sequence)
- **end**: the end time of the PIR sensor (relative to the start of the sequence)
- **name**: the name of the PIR sensor being activated (from the pir_locations list)
- **index**: the index of the activated sensor from the pir_locations

**Fig. 5:**
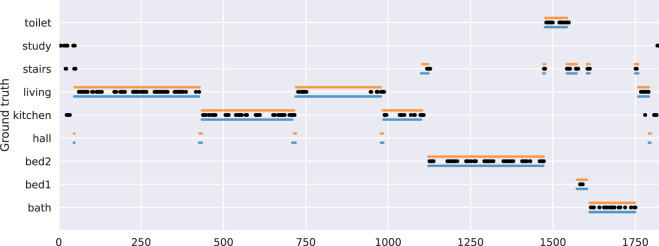
Example PIR for training record 00001. The black lines indicate the time duration where a PIR is activated. The blue and green wide horizontal lines indicate the room occupancy labels as given by the two annotators that labelled this sequence.

Example PIR sensor activation and ground truth locations are overlaid in Fig. 5. Note, the PIR sensors can be noisy as they are based on infra-red technologies, which can be affected by strong natural light.

#### pir.csv

This file contains the PIR data processed and sampled at a 0.1 second period (for raw view) or 1.0 seconds (for the sphere-challenge-2016 and sphere-challenge-complete-2022 views). The file consists of dense binary data for each PIR location (bath, bed1, bed2, hall, kitchen, living, stairs, study and toilet) and one supplementary column containing time.

#### acceleration.csv

The acceleration file consists of four columns:

- **t**: this is the time of the recording (relative to the start of the sequence)
- **x/y**/**z**: these are the acceleration values recorded on the x/y/z axes of the accelerometer.

**Fig. 6:**
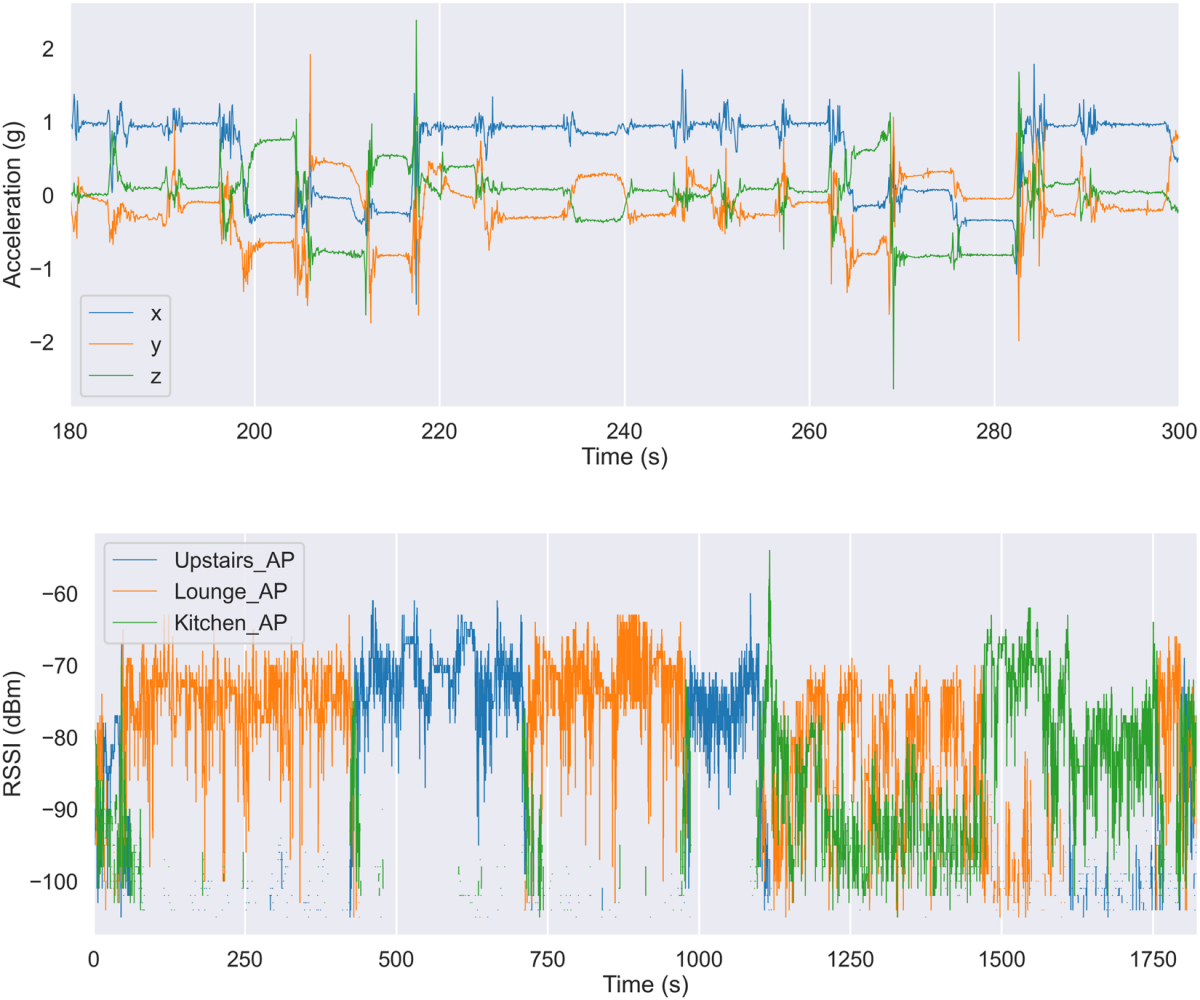
Example acceleration and RSSI signals for training record 00001. The line traces indicate the accelerometer/RSSI values recorded by the access points. The horizontal lines indicate the ground-truth as provided by the annotators (two annotators annotated this record, and their annotations are depicted by the green and blue traces respectively). (**Top**) Acceleration signal trace shown over a 5 minute time period. Annotations are overlaid. (**Bottom**) RSSI values from the four access points. Room occupancy labels are shown by the the horizontal lines.

Sample acceleration and RSSI with overlaid annotations are shown in Fig. 6. The top Fig. 6 shows the acceleration signals overlaid with activity labels.

#### rssi.csv

The RSSI file consists of five columns:

- **t**: this is the time of the recording (relative to the start of the sequence)
- **kitchen**/**living**/**study**/**stairs**: these specify the RSSI signal as received by each access point. Empty values indicate that the access point did not receive the packet.

The bottom Fig. 6 shows the RSSI signal information with the overlaid room occupancy.

#### rgbd_*.csv

The following sixteen columns are found in the rgbd_hallway.csv,

rgbd_kitchen.csv and rgbd_living.csv files:

- **t**: The current time (relative to the start of the sequence)
- **centre_2d_x**/**centre_2d_y**: The x- and y-coordinates of the centre of the 2D bounding box.
- **bb_2d_br_x/bb_2d_br_y**: The x and y coordinates of the bottom right (br) corner of the 2D bounding box
- **bb_2d_tl_x/bb_2d_tl_y**: The x and y coordinates of the top left (tl) corner of the 2D bounding box
- **centre_3d_x/centre_3d_y**/**centre_3d_z**: the x, y and z coordinates for the centre of the 3D bounding box
- **bb_3d_brb_x**/**bb_3d_brb_y**/**bb_3d_brb_z**: the x, y, and z coordinates for the bottom right back (brb) corner of the 3D bounding box
- **bb_3d_flt_x**/**bb_3d_flt_y**/**bb_3d_flt_z**: the x, y, and z coordinates of the front left top (flt) corner of the 3D bounding box.

**Fig. 7:**
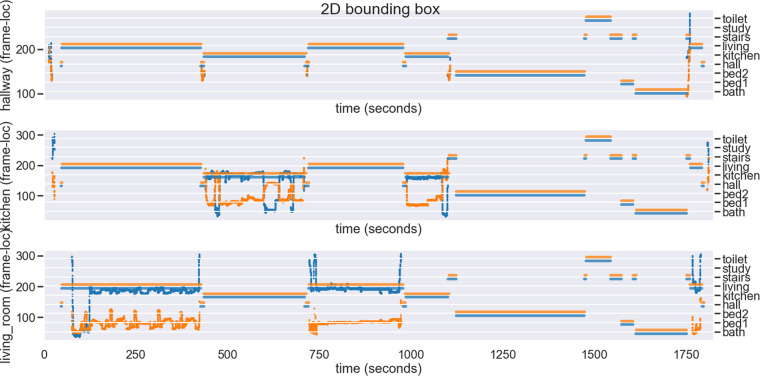
Example centre_3d for training record 00001. The horizontal lines indicate annotated room occupancy. The blue, green and red traces are the *x*, *y*, and *z* values for the 3D centre of mass.

Example 3D centre of mass data is plotted for the hallway, living room and kitchen cameras in Fig. 7. Room occupancy labels are overlaid on these, where we can see strong correspondence between the detected persons and room occupancy.

### Annotation data files

The following two sets of files need not be used for the challenge, but are included to facilitate users that wish to perform additional modelling of the sensor environment. For example, indoor localisation can be modelled with the locations.csv file^24^.

#### activity_*_raw.csv

This file provides the individual annotations as provided by the annotators. The target variables are the same as for assets/activity_labels.csv. The following 20 activities are annotated: {*a_ascend*, *a_descend*, *a_jump*, *a_loadwalk*, *a_walk*, *p_bent*, *p_kneel*, *p_lie*, *p_sit*, *p_squat*, *p_stand*, *t_bend*, *t_kneel_stand*, *t_lie_sit*, *t_sit_lie*, *t_sit_stand*, *t_stand_kneel*, *t_stand_sit*, *t_straighten*, *t_turn*}.

As before, the prefix ‘a_’ indicates an ambulation activity (i.e., an activity consisting of continuing movement), ‘p_’ annotations indicate static postures (i.e., times when the participants are stationary), and ‘t_’ annotations indicate posture-to-posture transitions.

The file activity_*_raw.csv contains the following four columns:

- **start**: the start time of the activity (relative to the start of the sequence)
- **end**: the end time of the activity (relative to the start of the sequence)
- **name**: the name of the label
- **index**: the index of the label name in activity_labels

#### location_*_raw.csv

This file provides the annotation labels for room occupancy. The same nine rooms are labelled as seen with the PIR sensor: {*bath*, *bed1*, *bed2*, *hall*, *kitchen*, *living*, *stairs*, *study*, *toilet*}.

The file assets/location_label.csv contains the following four columns:

- **start**: the time a participant entered a room (relative to the start of the sequence)
- **end**: the time the participant left the room (relative to the start of the sequence)
- **name**: the name of the room (from the location_labels list)
- **index**: the index of the room name starting at 0

#### activity.csv/location.csv

The activity.csv and location.csv files contains the aggregated ground-truth from multiple annotators on the activities and locations. As briefly introduced in the Methods section, the annotations are separated into a one-second time window. Within each window, the annotated length of each activity from each annotator is calculated, the final ground-truth is given as the normalised total annotated time for every activity (e.g., the rows sum to one).

The activity.csv files are dense CSV with columns corresponding to time and the labels listed in location_*_raw.csv. Similarly, the location.csv files are also in dense CSV format, but with columns corresponding to those in location_*_raw.csv.


per_ann_activity_*.csv/per_ann_location_*.csv


These files provide a dense binary CSV file of each annotator for activity and location. Averaging over the set of annotation files will produce the data in activity.csv/location.csv. The columns of per_ann_activity_*.csv are the activity labels from activity_*_raw.csv, and the columns of per_ann_location_*.csv are the location labels from location_*_raw.csv.

### Supplementary data files

#### meta.json

This file contains the metadata of the file including the sequence start and end times, the ID of the annotators as a list of integers, and the ID of the volunteer as an integer.

### Programatic loading

For convenience, two helper classes are provided to load data from raw.


from sphere_challenge import ProcessedSequence, RawSequence# Load a raw viewraw = ProcessedSequence.load_from_path(“data/raw/001”)# Load sphere-challenge-2016 testsc2016 = ProcessedSequence.load_from_path(“data/sphere-challenge-2016/test/00011”)# Load sphere-challenge-2016 testsc2022 = ProcessedSequence.load_from_path(“data/sphere-challenge-complete-2022/test/00011”)


The following member variables are exposed for RawSequence objects instantiated to a variable called obj:

- obj.meta: an object with additional member variables of start, end, annotators, user_id
- obj.acceleration: a Pandas dataframe with t (time), x, y, z columns
- obj.rssi: a Pandas dataframe with
- obj.pir: a Pandas dataframe with t, bath, bed1, bed2, hall, kitchen, living, stairs, study, toilet
- obj.rgbd: an object with additional fields of hall, kitchen, and living. Each sub-field is a Pandas dataframe with columns indicated in rgbd_*.csv.
- obj.labels.activity: a Pandas dataframe with the activity labels as columns (see activity_*_raw.csv)
- obj.labels.location: a Pandas dataframe with the location labels as columns (see location_*_raw.csv)

Alternatively, to load the raw data from file, one may call RawSequence.load_from_path in the same manner as above.

## Technical Validation

In this section we provide a set of technical validation results on the dataset. As mentioned above, the overall aim of the sensor platform is to capture different behaviour patterns that are potentially linked to certain health conditions. As the sensors are equipped within residential homes, people’s behaviours can hence be quantified through these three variables: time, location, and activity. In this section, we demonstrate the dataset via a fundamental application: activity recognition with supervised machine learning.

**Fig. 8:**
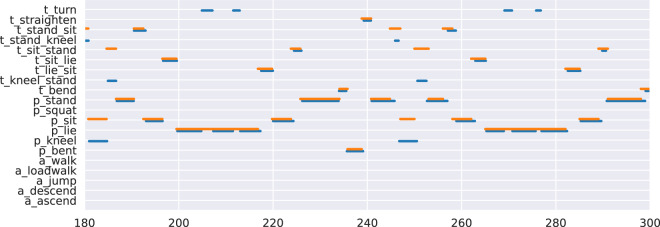
The annotated activities of one of the scripts. (two annotators annotated this record, and their annotations are depicted by the red and blue traces respectively).

Since for each scripted experiment we allocated two annotators to provide the ground-truth for the activities, there can potentially be disagreements between the two annotators at a certain time point. On the other hand, for a given time range (e.g., one second), it might contain multiple annotations according to the original precision. Figure 8 shows an example from two annotators on a particular script. As indicated, while both annotators agree on most activities, some styling differences can still be observed. For instance, one of the annotators records the activity of consecutive jumping with three segments, but the other annotator treats them together as one. With such differences in mind, we formalised the annotations into a normalised vector as a frequentist view of probabilistic classification. For a given second, we calculate the overall time length given by all the annotators, then normalise these time intervals into a probability vector over all the activities/locations.

**Fig. 9:**
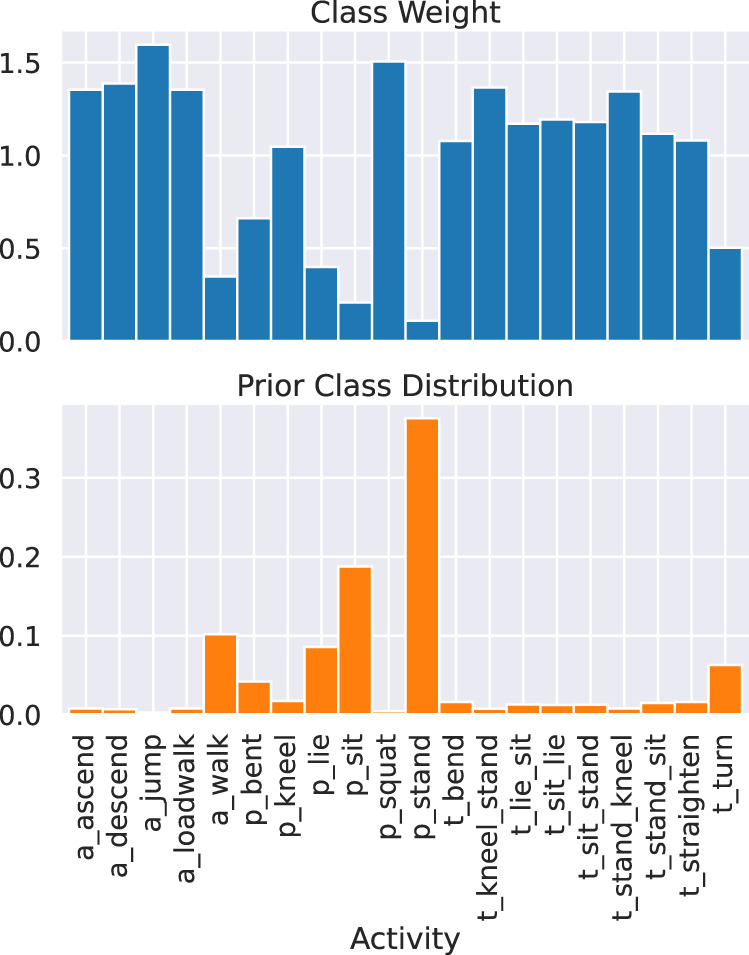
The class distribution of the annotated activities (top). The associated class weights for Brier score (bottom).

**Fig. 10:**
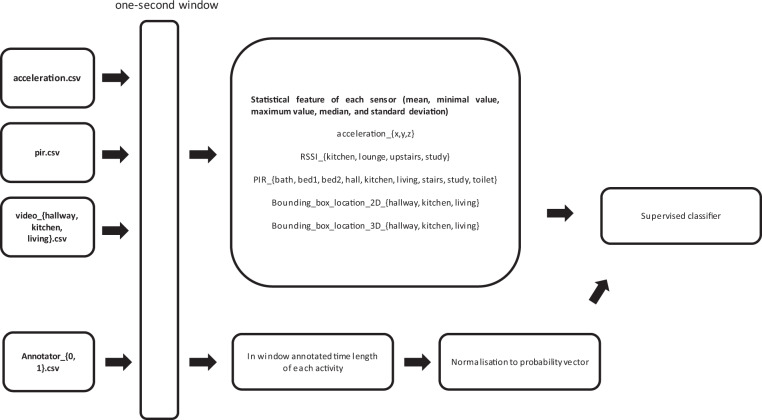
The flowchart of the data (sensor readings and annotations) processing steps for supervised classifier training (best viewed zoomed in).

Here we demonstrate a simple approach to achieve the task described above, which uses some basic features together with a probabilistic nearest neighbour classifier, with the implementation provided by the scikit-learn package^25^. For the features, we extract the mean, minimal value, maximum value, median, and standard deviation from each modality (Acceleration and RSSI values from the wearable device, PIR sensor values, bounding box locations from the three camera locations.), and combine them into a feature vector for that particular second. After obtaining the features, a probabilistic nearest neighbour can be used to model the probability of each activity on a given second, based on a number of closest feature vectors. The overall steps are shown by a flowchart in Fig. 10.

**Fig. 11:**
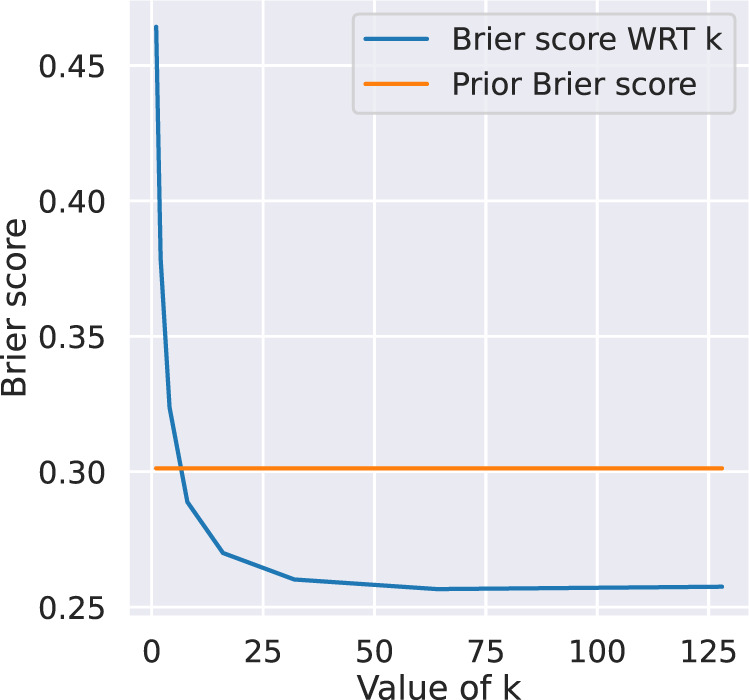
The effect of the number of neighbours, evaluated by the Brier score. The performance is obtained by leave-one-out validation (train with sequence 01 to 09, tested on sequence 10).

**Fig. 12:**
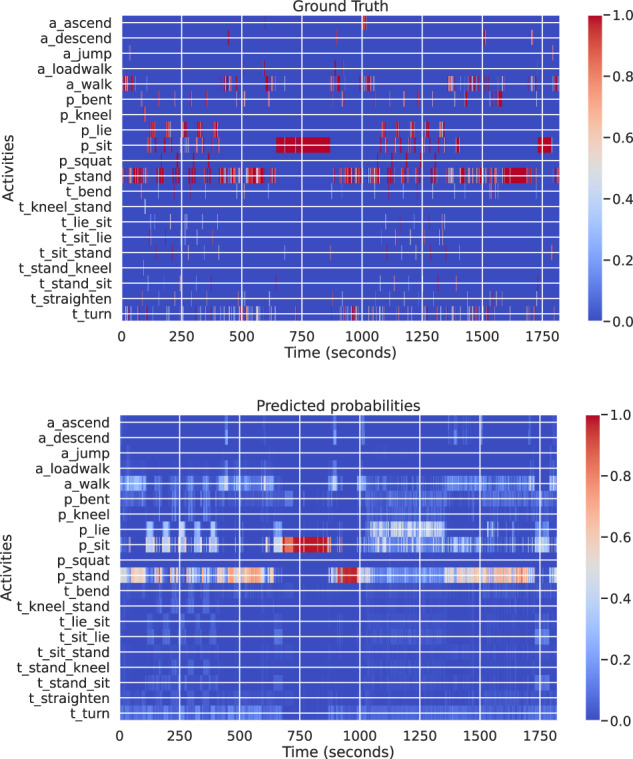
(**Top**) The ground-truth of a given sequence. (**Bottom**) Predictions from a probabilistic nearest neighbour classifier. The heatmap reflects the probability (red being 1 and yellow being 0).

Figure 12 shows the results on a particular scripted experiment, while the model is trained on other nine further experiments (using 128 nearest neighbours). As indicated by the figure, a simple model described above is able to capture the major activities like stand and sit, while other minor activities and postures can be still improved by considering advanced feature and model learning approaches.

In terms of performance, since both the ground-truths and model predictions are probability vectors, one of the most common evaluation measures here is the Brier score, which equals to the sum of squared errors among the probability vectors^26,27^. Since the class distribution is highly unbalanced in this dataset (e.g., the activity of jump only takes a few seconds while the posture of standing can take minutes), it is common to further adjust the weights of the Brier score of each class as in the case of cost-sensitive classification^27^. Here we consider to set the weights of each class as the reciprocal of their marginal class probability value. An example is given in Fig. 9, and we can see that minority classes such as ‘jump’ and ‘squat’ are assigned top weights for the score evaluation.

Figure 11 shows the Brier score on different number of neighbours considered by the model described above. For this particular task, it can be seen the performance increases when the number of neighbours grows larger. While the models are very simple in this case, it is sufficient to demonstrate the datasets are of great value when modelling the activities within a residential home.

## Usage Notes

In this section we provide some additional notes for this dataset, as well as introducing other related work on health-care and sensor-based behaviour modelling. Diethe *et al*.^7^ provides a general view of the SPHERE project in terms of applied machine learning and data mining. Diethe *et al*.^28^ introduces HyperStream, which is a generic stream processing software developed under the SPHERE project. Diethe *et al*.^29^ discusses different approaches to fuse multi-modal streams towards smart home applications. Various approaches on activity recognition have been further documented^19,30,31^, generating discussion of some of the results on monitoring long-term health conditions^32,33^.

## Data Availability

A git repository is publicly available at https://github.com/IRC-SPHERE/sphere-challenge-sdata/. In this repository a number of scripts for visualisation, bench marking and data processing are available. (All subsequent sensor images were generated using these scripts).
